# Formulation and characterisation of alginate hydrocolloid film dressing loaded with gallic acid for potential chronic wound healing

**DOI:** 10.12688/f1000research.52528.1

**Published:** 2021-06-07

**Authors:** Jhing-Ee Gan, Chai-Yee Chin

**Affiliations:** 1 School of Pharmacy, Faculty of Health and Medical Sciences, Taylor's University, Subang Jaya, Selangor, 47500, Malaysia; 2 Centre for Drug Discovery and Molecular Pharmacology (CDDMP), Taylor’s University, Subang Jaya, Selangor, 47500, Malaysia

**Keywords:** Alginate, pectin, gallic acid, wound dressing, hydrocolloid film

## Abstract

**Background: **A dramatic growth in the prevalence of chronic wounds due to diabetes has represented serious global health care and economic issues. Hence, there is an imperative need to develop an effective and affordable wound dressing for chronic wounds. Recent research has featured the potential of bioactive compound gallic acid (GA) in the context of wound recovery due to their safety and comparatively low cost. However, there is a scarcity of research that focuses on formulating GA into a stable and functional hydrocolloid film dressing. Thus, this present study aimed to formulate and characterise GA-loaded alginate-based hydrocolloid film dressing which is potentially used as low to medium suppurating chronic wound treatment.

**Methods: **The hydrocolloid composite films were pre-formulated by blending sodium alginate (SA) with different combinations of polymers. The hydrocolloid films were developed using solvent-casting method and the most satisfactory film formulation was further incorporated with various GA concentrations (0.1%, 0.5% and 1%). The drug-loaded films were then characterised for their physicochemical properties to assess their potential use as drug delivery systems for chronic wound treatment.

**Results:** In the pre-formulation studies, sodium alginate-pectin (SA-PC) based hydrocolloid film was found to be the most satisfactory, for being homogenous and retaining smoothness on surface along with satisfactory film flexibility. The SA-PC film was chosen for further loading with GA in 0.1%, 0.5% and 1%. The characterisation studies revealed that all GA-loaded films possess superior wound dressing properties of acidic pH range (3.97-4.04), moderate viscosity (1600 mPa-s-3198 mPa-s), optimal  moisture vapor transmission rate (1195 g/m ^2^/day, 1237g/m ^2/^day and 1112 g/m ^2^/day), slower moisture absorption and film expansion rate and no chemical interaction between the GA and polymers under FTIR analysis.

**Conclusion:** An SA-PC hydrocolloid film incorporated with gallic acid as a potentially applicable wound dressing for low to medium suppurating chronic wounds was successfully developed.

## Introduction

Delayed wound healing that leads to chronic diabetic foot ulcers (DFU) and lower limb complications are the most devastating complications of diabetes which has impacted 40 to 60 million people worldwide ( International Diabetes Federation, 2019). In the year of 2001, the United States (US) healthcare system reported an expenditure of 10.9 billion USD in management and treatment of DFU. The expenditure of wound management for individuals with diabetes and foot ulcer are 5.4 times higher in the year of the first event and 2.6 times higher in the year of the second event as compared to individuals with diabetes but without foot ulcers. DFU can lead to serious infection, gangrene, foot amputation and even death if proper care and attention was not given. Wound healing treatment is more challenging specifically in persons with DFU as the delayed wound healing is contributed by multiple mechanisms such as the reduced response of cells and growth factors resulting in reduced peripheral flow of blood and local angiogenesis. Therefore, wound care is a crucial component of DFU management ( Brem & Tomic-Canic, 2007).

Among the plethora of modern wound dressings present in the marketplace nowadays, moisture-retentive dressings such as hydrocolloid films have advanced to become a famous wound healing modality for moist wound management. Upon contact with the wound exudate, this matrix converted to a gel sheet from a dry dressing to create moist surroundings around the wound area to facilitate the proliferation and migration of dermal fibroblasts as well as to advance collagen synthesis which is beneficial in reducing the formation of scar ( Moura *et al*., 2013). Sodium alginate (SA) is a hydrophilic soluble salt of alginic acid, which is a polysaccharide that exists in the cell wall of brown algae. It possesses good film fabrication properties and is widely used as a controlled release medium in delivering drugs with its ability to expand itself upon water absorption. Pectin (PC), a linear polysaccharide which has been explored extensively for film dressings application due to several advantages in wound healing discovered namely, its (1) hydrophilicity, which facilitates exudate removal in wounds by reacting with the wound fluid to create a gel with soft texture over the wound site, (2) role as binding agent when added to wounds to safeguard the growth factors from degradation hence promote new cell generation, and (3) ability to retain an acidic environment of the wound to prevent any microbial growth ( Munarin *et al*., 2012). Given their safety and relatively affordability, there has been a gradually increasing interest in using bioactive plant compound in the context of wound healing. One such extract is gallic acid (GA), which is a natural phenolic compound found in the fruits, leaves, and wildflowers ( Badharu *et al*., 2015). GA had been well demonstrated for antioxidant, analgesic, anti-inflammatory, including anti-diabetic properties. Singh *et al*. (2019) and Kaparekar *et al*. (2020) have noted an improved wound contraction as well as a reduction in duration of re-epithelialization of the excision wound with the use of GA. In short, all the beneficial features that GA possess to support its advancement into a practical wound recovery agent. This present study aimed to formulate and characterise GA-loaded alginate (SA) based hydrocolloid film dressing which is potentially used as low to medium suppurating chronic wound treatment.

## Methods

### Materials

Gallic acid 97.5-102.5% (CAS No. 149-91-7, MW: 170.12g/mol), sodium carboxymethylcellulose (CAS No. 9004-32-4) and Pluronic F-127 (CAS No. 9003-11-6) were supplied by Sigma Aldrich (USA). Sodium Alginate (CAS No. 9005-38-3, MW~ 20-40 kDa) was procured from Fisher Scientific (UK), whereas pectin pure (CAS No. 9005-69-5, >99.0%) was supplied by Sime Scientific.

### Formulation of gallic acid composite film dressings

Five (5) different combinations of polymers were used in the pre-formulation of blank hydrocolloid film which include: sodium alginate (SA), pectin (PC), sodium carboxymethylcellulose (NaCMC), chitosan (CS) and Pluronic F-127 (PF127) presented in Table 1. The blank hydrocolloid films were solvent-casted by filling the uniform gel slurry (20g) into petri dishes (d = 90 mm) then dried in the oven (39 ± 3°C) for 24 h. After the preliminary evaluation, GA was loaded into the sodium alginate pectin (SA-PC) composite film to produce the drug-loaded film as summarized in Table 2. Formulation of GA-loaded films with the strength of 0.1, 0.5 and 1.0% w/v were based on literature review and formulation suitability. The dried film was stored in desiccators to prevent it from reacting with moisture from humidity.

**Table 1.** Composition of drug-free hydrocolloid films which contain composite polymers.

Film composition	SA	PC	SA-PC	SA-CS	PC-CS	NaCMC	SA-NaCMC	PC-NaCMC	CS	NaCMC-CS	PF-127	SA-PF127	PC-PF127
**Sodium alginate (g)**	6	6	3	3	-	6	3	-	-	-	-	3	-
**Pectin (g)**	-	-	3	-	3	-	-	3	-		-	-	3
**NaCMC (g)**	-	-	-	-	-	-	3	3	-	3	-	-	-
**Chitosan (g)**	-	-	-	3	3	-	-	-	6	3	-	-	-
**Pluronic F-127 (g)**	-	-	-	-	-	-	-	-	-	-	6	3	3
**Glycerol (ml)**	3	3	3		3	3	3	3	3	3	3	3	3
**Distilled water (ml)**	91	91	91		91	91	91	91	91	91	91	91	91

* Abbreviations: SA- Sodium alginate, PC-Pectin, NaCMC-Sodium carboxymethylcellulose, CS-Chitosan, PF127-Pluronic F-127.

**Table 2.** Composition of sodium alginate hydrocolloid films containing various strengths of gallic acid (GA).

Constituents	0.1% w/v	0.5% w/v	1.0% w/v
**Sodium alginate (g)**	3	3	3
**Pectin (g)**	3	3	3
**Gallic acid (g)**	0.1	0.5	1.0
**Glycerol (ml)**	3	3	3
**Distilled water (ml)**	90.9	90.5	90

### Physical assessment and microscopic analysis

The morphology of film samples was examined under a polarized microscope built with a camera. The films were put on top of a glass slide with the slip covered before viewing under the microscope at a magnification of 4x. The snapshots of the magnified film were captured under bright and polarized light.

### pH study and viscosity profiles

Digital pH meter (Sartorius pH meter, Germany) was used to determine the acidity properties of polymers (before drying). The viscosity of polymers (before drying) was determined by using a rheometer (Brookfield DV2T Viscometer, USA). Gel samples were sheared continuously at a rate of 100 rpm for I min with spindle LV-4 (64) or 200rpm for one min with spindle LV-3 (63).

### Fourier transform infrared (FTIR) analysis

Fourier transform infrared (FTIR) analysis was executed with a spectrophotometer (Perkin Elmer Spectrum 100 FTIR Spectrometer, USA) equipped with an attenuated total reflection (ATR) assembly. The FTIR spectra of the pure gallic acid, SA, PC and drug-loaded films were recorded.

### Moisture vapour transmission rate (MVTR)

The films (diameter = 13mm) fitted over the brim of 20 mL clear glass vials with dry silica beads (2 g). The vials containing the film with dry silica beads, were weighed as 0 h and kept in a moist chamber at an RH of 88 ± 2% at 33°C. The vial was taken out from the desiccator to perform weighing at every 1 h interval for the first subsequent eight hours then followed by the 24 ^th^ hour. The weight gained for respective hours were reported and repeated in triplicate to obtain the average values. The MVTR was calculated by using Eq. (1):

Moisture vapour transmission rate (MVTR)=W/S (1)

where, *W* is the weight that is gained by desiccant over 24 h, and *S* is the exposed surface area of the film (m ^2^); MVTR expressed in units as g/m ^2^/day.

### Moisture absorption

The film samples (diameter = 22mm) kept in desiccators with silica beads overnight to retain their dryness. Weighing boats (small) were weighed and labeled accordingly with the film samples. Its initial weight at 0 ^th^ hour was regarded as zero weight gained. The trimmed films were transferred into a moist chamber (RH 88 ± 2%). Weighing boats with the film samples were then weighed again at the 24 ^th^ hour as final weight gained. The moisture absorption was obtained using Eq. (2):

$$\mathrm{Moisture}\;\mathrm{absorption}\;\left(\%\right)=\left[(W_{t}-W_{o}\right)/W_{o}]\times 100\%$$ (2)

where, *W*_*t*_ is the weight of film samples after 24 h, and *W*_*o*_ is the weight of film sample before inserted into the moist chamber.

### Swelling study

Film swelling was studied using a gelatin model adapted from a previously established protocol by Chin *et al*. (2018). The films (diameter = 22 mm) placed at the center of gelatin gel contained in petri dishes. The diameter of films was measured using vernier calipers at the beginning of the experiment as 0 h. Next, the differences in film expansion were reported at a predetermined time interval of every 1 h in the subsequent first 8 hours and after the 24 hours. Each measurement of film was carried out three times and the average values of expansion ratio was calculated with the Eq. (3):

$$\mathrm{Film}\;\mathrm{expansion}\;\mathrm{ration}\left(\%\right)=\left[\left(D_{r}-D_{o}\right)/D_{o}\right]\times 100\%$$ (3)

where *D*_*t*_ is the diameter of film after expansion and *D*_*o*_ is the diameter of film before expansion.

## Results

All the gel samples were sheared continuously at a rate of 100 rpm for 1 min with spindle LV-4 (64) except the asterisks (*) represent gel samples that sheared continuously at a rate of 200 rpm for one min with spindle LV-3 (63).

## Discussion

**Figure 1.**
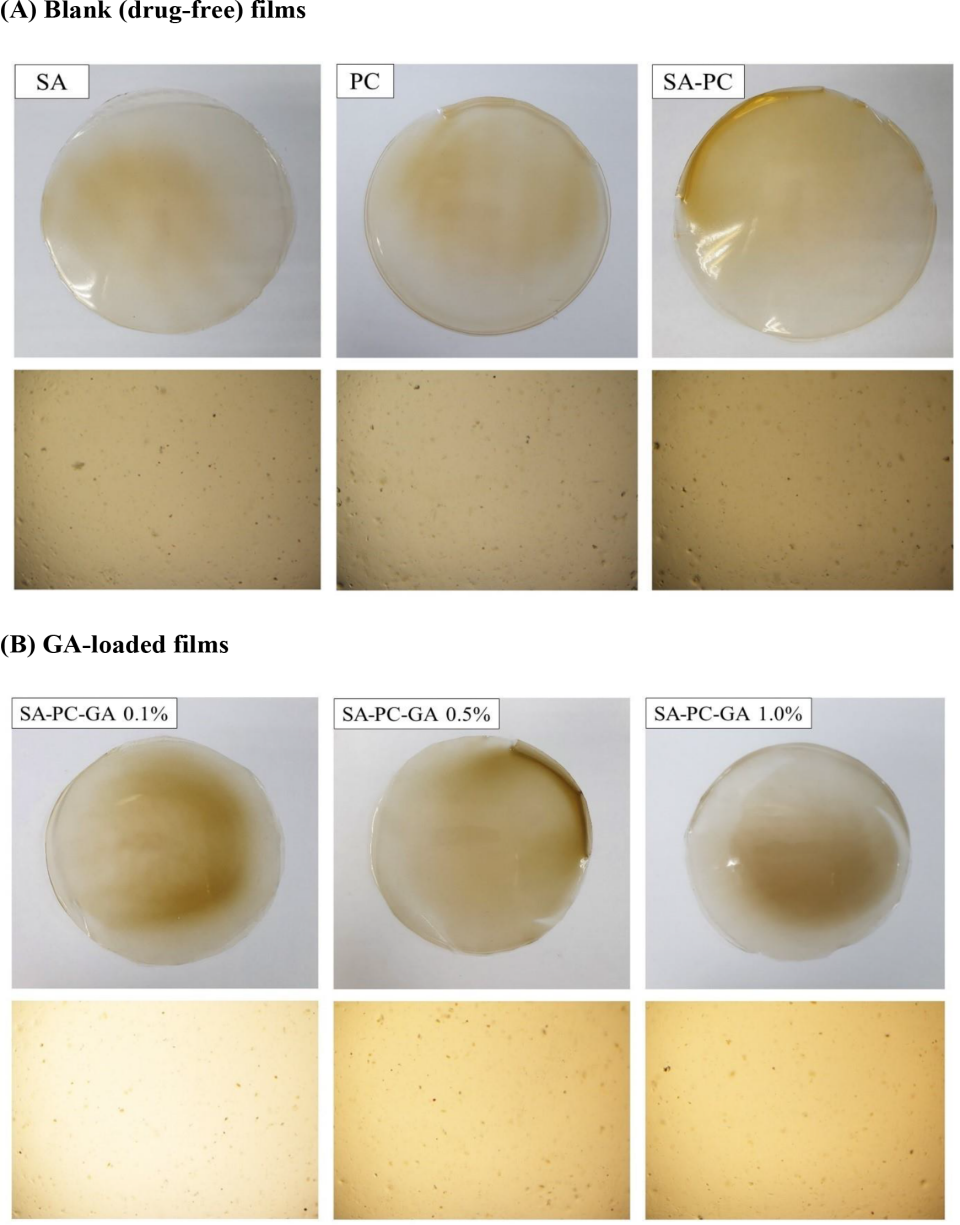
Macroscopic and microscopic images of (A) SA, PC and blank SA-PC hydrocolloid films and (B) Gallic acid-loaded films of various strengths (SA-PC-GA 0.1%, 0.5% and 1.0% w/v) taken on physical examination and under brightfield microscope at 4× magnification.

Pre-formulation study is an essential preliminary study to ensure the successful establishment of an optimum drug delivery system prior to formulation of a novel modality for wound healing. The SA-PC composite films appeared as translucent, smooth texture without air bubbles when viewed under bright light microscope along with adequate flexibility in texture. Films formulated with SA and PC alone, also shown as translucent with slightly uneven surface and has a reduced flexibility in nature as compared to SA-PC composite films ( Figure 1A). All in all, SA-PC films were identified as the most satisfactory film and selected for further loading with GA of various strengths (0.1%, 0.5%, 1.0% w/v) and were continued for characterisation studies. All the films showed homogenous and smooth surface without any visible bumps, flaws or cracks when viewed under the microscope hence indicating excellent compatibility between GA and SA-PC polymers ( Figure 1B).

All the hydrocolloid gel slurry had exhibited acidic pH values, as shown in Table 3, except for the formulations produced with chitosan which are the NaCMC-CS and SA-CS. All the polymeric gels formulated using PC polymer generally displayed a slightly lower pH value range (3.56-5.23) than the other combination of polymers having pH value range (6.18-8.19), with PC polymer alone having the lowest pH value of 3.74. All the mixtures of SA-PC polymeric gel loaded with GA also exhibited acidic properties with pH value range (3.97-4.04) as shown in Table 4. Therefore, hydrocolloid film with slight acidic properties formulated in this study might promote wound recovery attributed to the role of pH in affecting the phases of wound repair.

**Table 3.** The pH and viscosity of drug-free polymeric gel (mean ± SD, n = 3).

Film composition	SA	PC	SA-PC	SA-CS	PC-CS	NaCMC	SA-NaCMC	PC-NaCMC	CS	NaCMC-CS	PF-127	SA-PF127	PC-PF127
pH	6.35 ± 0.04	3.74 ± 0.06	5.05 ± 0.07	7.66 ± 0.07	4.51 ± 0.07	6.20 ± 0.06	6.72 ± 0.03	5.23 ± 0.1	-	8.19 ± 0.06	6.86 ± 0.06	6.18 ± 0.06	3.56 ± 0.07
Viscosity (mPa-s)	5396 ± 307.72	326 ± 15.10	1974 ± 21.63	726 ± 15.87	58 ± 3.46	-	-	-	-	-	3.8 ± 1.39*	594 ± 6.00*	86 ± 3.46*

All the gel samples were sheared continuously at a rate of 100 rpm for 1 min with spindle LV-4 (64) except the asterisks (*) represent gel samples that sheared continuously at a rate of 200 rpm for 1 min with spindle LV-3 (63).

**Table 4.** The pH and viscosity of polymeric gel loaded with various strengths of gallic acid (mean ± SD, n = 3).

Constituents	0.1% w/v	0.5% w/v	1.0% w/v
pH	4.04 ± 0.04	4.04 ± 0.04	3.97 ± 0.03
Viscosity (mPa-s)	1600 ± 9.17	2300 ± 104.33	3198 ± 414.52

The viscosity of hydrocolloid film is crucial as it determines the formulation stability and efficacy in terms of drug release (Matthews et. al, 2006). Polymeric gel of PC, SA-CS, PC-CS, PF-127, SA-PFl27 PC-PFl27 possess a viscosity value ranging from 58 to 726 mPa-s which is too low for the formulated film to retain on wound area for sufficient time for wound healing. On the contrary, SA polymeric gel possesses a high viscosity value of 5396 mPa-s, hence causing the formulated film to have less flexibility. The formulated NaCMC hydrocolloid gel possesses overly high viscosity above 6000 mPa-s and tend to produce excessively hard texture films. Therefore, SA-PC with moderately high viscosity value of 1974 mPa-s has been selected to further incorporation with GA. All GA-loaded SA-PC hydrocolloid films were displayed high viscosity and the viscosity increased from 1600, 2300 to 3198 mPa-s as the concentration of drug incorporated increases. Zaman *et al.* (2011) also presented the findings that the greater the viscosity of a developed film, the better it is to maintain the integrity as a drug delivery system.

**Figure 2.**
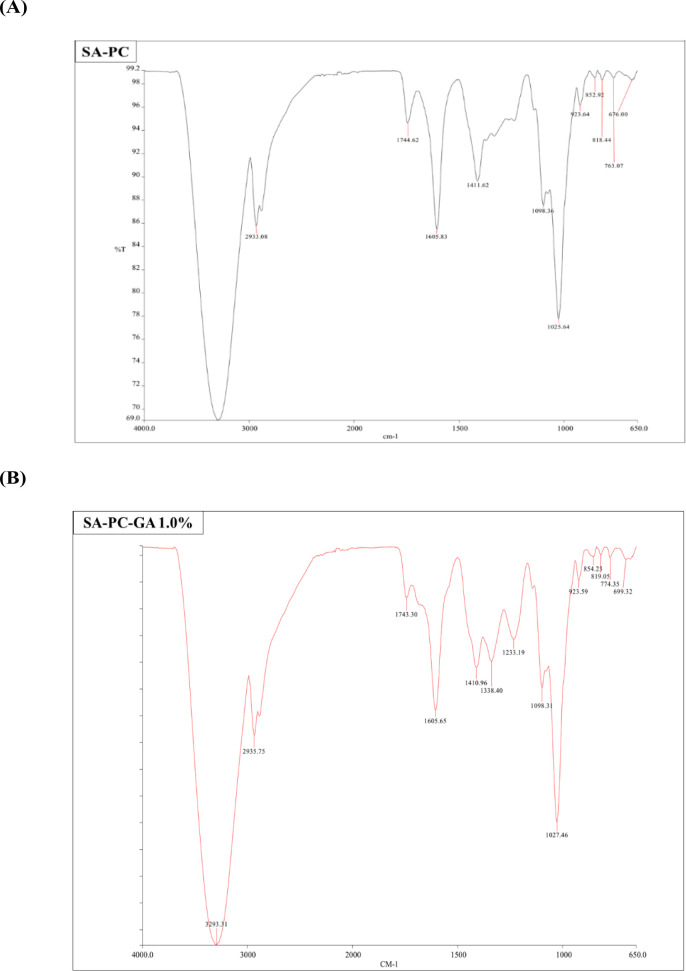
FTIR spectra of (A) Blank SA-PC film, (B) Gallic acid-loaded film (SA-PC-GA 1.0%). * Abbreviations: SA, sodium alginate; PC, pectin; GA, gallic acid.

FTIR was conducted to identify any chemical interactions and investigate the compatibility between GA with the polymers within the film. The FTIR spectra of blank and GA-loaded films are shown in Figure 2A & 2B. GA characteristic peaks at 3280 and 3493 cm ^-1^ suggested to be the aromatic and carboxylic O—H stretching, the hydroxyl groups (—OH) present in positions three, four, and five of the aromatic rings. Bands at 1695 cm ^-1^ are for the C=O stretching of carboxylic acid whereas bands at 1540 and 1468 cm ^-1^ areas corresponding to C=C aromatic stretching vibration. The FTIR spectra of SA-PC-GA films exhibited characteristic peaks of individual polymers of SA and PC, as well as the presence of the prominent peaks of GA. Moreover, the FTIR spectra of drug-free and GA-loaded films did not display any major distinction from each other, indicating the lack of obvious chemical interaction occurred between the polymers and the drug. In short, the bioactive constituent did not lose its activity when loaded into the films by blending with SA‐PC polymers.

**Figure 3.**
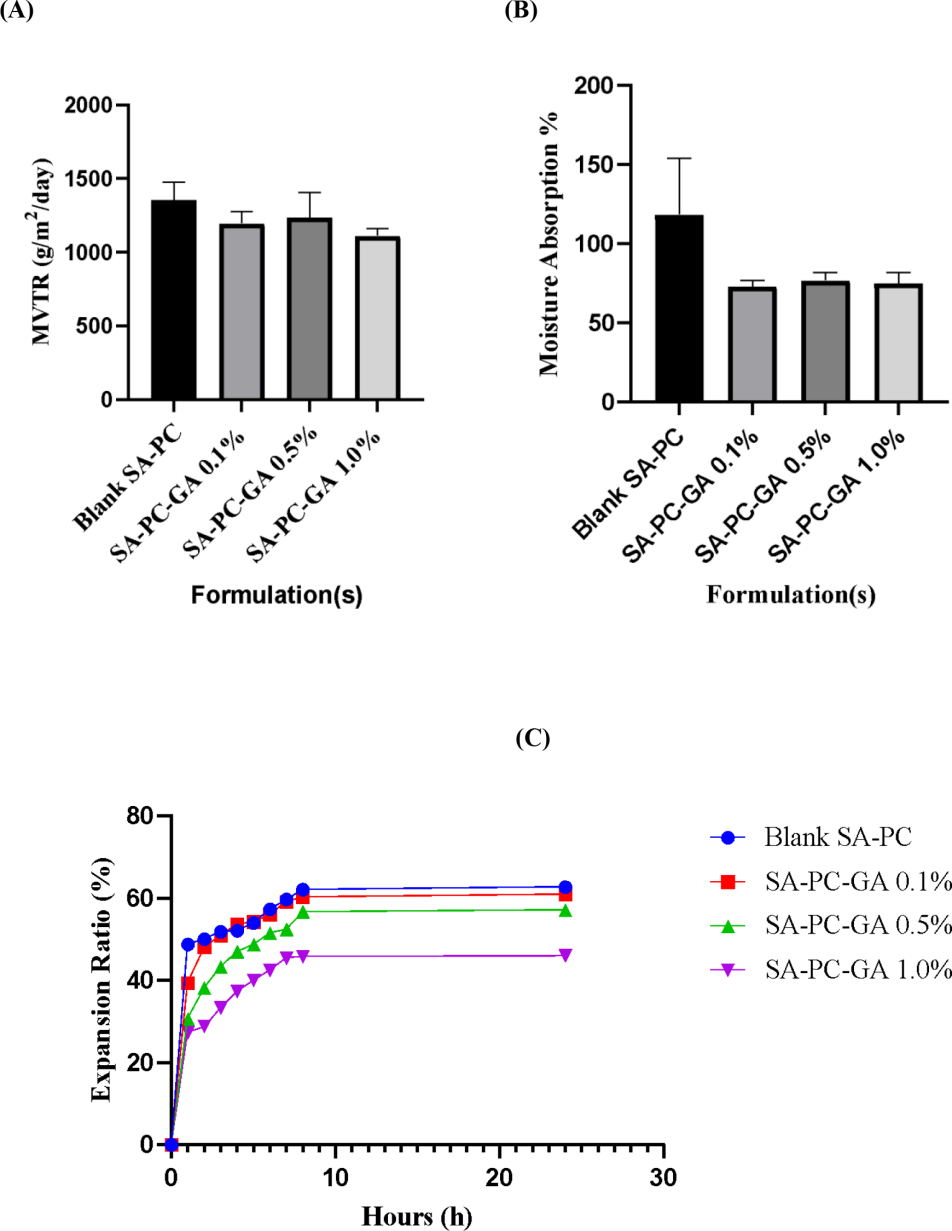
(A) Moisture vapor transmission rate (MVTR), (B) Moisture absorption % and (C) Expansion ratio (%) of film formulations incorporated with gallic acid (mean ± SD, n = 3).

A desirable wound dressing should provide an optimal moisture vapor transmission rate (MVTR) which is crucial for creating a moist environment to advance wound recovery ( Bajpai *et al*., 2014). According to Gupta *et al*. (2010), MVTR for normal skin is 204 g/m ^2^/day whereas for wounded skin, it can range from 279 to 5138 g/m ^2^/day. As shown in Figure 3A, the MVTR for all the formulated films (0.1%, 0.5% and 1.0% w/v SA-PC-GA) were reported to fit within the range for injured skin, which is 1195, 1237 and 1112 g/m ^2^/day with no significant differences (p>0.05) between the films and blank SA-PC film. It has also been revealed that the moisture vapor gained per area of films has increased gradually over the first 8 h of experiment to facilitate an optimum gaseous exchange for wound healing. This is in accordance with the findings reported by Xu *et al.* (2016) that the MVTR within ≤ 2028 g/m ^2^/day is appropriate to create a moist wound bed. In short, all the formulated films loaded with GA able to maintain sufficiently high MVTR for accelerating epithelialization process at injured wound area ( Tan *et al*., 2020). The moisture absorption ratio of GA loaded hydrocolloid films ranged from 73.05-76.87%, as displayed in Figure 3B with no significant difference (p > 0.05). The absorption of moisture by the films could be assignable to the constituents that made up the film, for instance glycerol. As reported by Rusli (2017), glycerol is capable of increasing the moisture absorption ability to a certain extent and causing an increment in thickness by film swelling. Therefore, it can be deduced that the GA-loaded films with a lower percentage of moisture absorption is more favorable for low to medium exudative wounds.

The swelling study was assessed to investigate the expansion rate of the hydrocolloid film dressing under exudative wound condition. As displayed in Figure 3C, the SA-PC-GA 0.1% hydrocolloid film presented with the highest hydration and expansion rate in the first 4 h. The film expansion ratio was presumed to be related to the viscosity profile of the formulated films, as reported by Thu *et al*. (2012). Since SA-PC-GA 0.1% w/v hydrocolloid film is having the least viscosity, it swells upon moisture uptake more rapidly and loses its circular shape quicker. The film expansion ratio was observed to be slower in SA-PC-GA 0.5% w/v and followed by SA-PC-GA 1.0% w/v hydrocolloid film, which is having the lowest expansion ratio. All the hydrocolloid films attained a nearly plateau state after 8 h, where they became highly viscous and could not expand further. In short, a wound dressing that can maintain its shape and structure for a prolonged period of application is desirable for highly exudative wounds. From this present study, it was postulated that SA-PC-GA 0.1% w/v hydrocolloid film is more suitable to be used for low suppurating wounds or shorter-term application. On the other hand, SA-PC-GA 1.0% w/v hydrocolloid film may be particularly useful in medium to heavy exudative wounds or longer-term application owing to its high-water retention capacity.

## Conclusion

In this present study, the GA-loaded hydrocolloid films exhibited ideal wound dressing properties of (1) acidic pH, (2) moderate viscosity, (3) absence of chemical interaction between drug and polymer excipients, (4) optimal MVTR, (5) lower moisture absorption, and (6) film expansion rate, thus suggesting its potential use as a wound dressing for low to medium suppurating wounds. Future investigations which focus on the in vivo performance of gallic acid loaded film formulations are necessary to establish its safety and efficacy profile as a chronic wound healing application.

## Data availability

### Underlying data

Harvard Dataverse: Formulation and Characterisation of Alginate Hydrocolloid Film Dressing Loaded with Gallic Acid for Potential Chronic Wound Healing. https://doi.org/10.7910/DVN/1AIBEU ( Gan and Chin *,* 2021) This project contains the following underlying data:

- Moisture Absorption Studies.csv
- Moisture Vapour Transmission Rate Studies.csv
- Swelling Studies.csv
- Microscopy images for all drug-free films and GA loaded hydrocolloid films
- pH and viscosity readings for all drug-free polymeric gels
- pH and viscosity readings for all GA-loaded polymeric gels
- Pure gallic acid FTIR image

Data are available under the terms of the Creative Commons Zero “No rights reserved” data waiver (CC0 1.0 Public domain dedication).
