# Supplementary material for: Formulation and characterisation of alginate hydrocolloid film dressing loaded with gallic acid for potential chronic wound healing
Source: F1000Res. 2021 Jun 7;10:451. [Version 1] doi: 10.12688/f1000research.52528.1 (PMC8237367; doi:10.12688/f1000research.52528.1)
Supplement: Peer review history

# Peer review: Reviewer response for version 1

**DOI:** 10.5256/f1000research.55822.r86941

**Published:** 2021-06-25

**Authors:** Azad Abdul Kalam

**Affiliations:** 1 Advanced Drug Delivery Laboratory, Pharmaceutical Technology Department, Faculty of Pharmacy, International Islamic University Malaysia, Kuantan, Malaysia

I am very happy to announce that this invited research paper has been completed in a very fluent language and in a proper manner. I read the research paper very carefully and realized that the research has practical application and novelty which enables the reader and other researchers to take their research work one step further and get the right direction.

 Even then I came to realize that the research work could be much more informative and meaningful if the author's will able to include drug (GA) entrapment and release study data. Thank you so much.

- The work clearly and accurately presented, and the authors have cited the recent published literature.
- I have found this study in appropriate design with its academic merit.
- Authors have described with sufficient details of methods and analysis provided to allow replication by other researchers for further investigation.
- I noticed that the statistical analysis and its interpretation is appropriate and scientific manner.
- In these current results described by authors data underlying the results available to ensure full reproducibility.
- The conclusions drawn adequately supported by the results presented in this manuscript.

Is the work clearly and accurately presented and does it cite the current literature?

Yes

If applicable, is the statistical analysis and its interpretation appropriate?

Yes

Are all the source data underlying the results available to ensure full reproducibility?

Yes

Is the study design appropriate and is the work technically sound?

Yes

Are the conclusions drawn adequately supported by the results?

Yes

Are sufficient details of methods and analysis provided to allow replication by others?

Yes

Reviewer Expertise:

My area of research is natural drug product development, advanced drug delivery system or develop drug delivery carrier.

I confirm that I have read this submission and believe that I have an appropriate level of expertise to confirm that it is of an acceptable scientific standard.

# Peer review: Reviewer response for version 1

**DOI:** 10.5256/f1000research.55822.r86942

**Published:** 2021-06-21

**Authors:** ‪Pijush Kumar Paul‬

**Affiliations:** 1 Department of Pharmacy, Gono Bishwabidyalay, Dhaka, Bangladesh

The manuscript describes the study of formulation and characterization of gallic acid-loaded alginate-based hydrocolloid film dressing for chronic wound treatment.

**Comments**

 The manuscript is well written however, additional proofreading of the manuscript’s English grammar could enhance the clarity of the paper.

 This manuscript does not do justice to the previous work. The review concerning the recent trends on wound management has not been cited.

- E.g., Okur, M. E., Karantas, I. D., Şenyiğit, Z., Üstündağ Okur, N., & Siafaka, P. I. (2020). Recent trends on wound management: New therapeutic choices based on polymeric carriers. *Asian journal of pharmaceutical sciences*, *15*(6), 661–684 ^1^.

The source of pectin should be written clearly including the country of origin. The source of chitosan is not mentioned in the materials section.

 There should be error bar in the figure 3.

 There are a few typos in the manuscript.

- E.g., on page 3, there will be 1 instead of I in the last line of pH study and viscosity profile of method section and there will be space between 200 and rpm. There will be space between 22 and mm in the first line of moisture absorption. In addition, there should be h instead of hour in the same paragraph.

Is the work clearly and accurately presented and does it cite the current literature?

Partly

If applicable, is the statistical analysis and its interpretation appropriate?

Partly

Are all the source data underlying the results available to ensure full reproducibility?

Yes

Is the study design appropriate and is the work technically sound?

Yes

Are the conclusions drawn adequately supported by the results?

Yes

Are sufficient details of methods and analysis provided to allow replication by others?

Yes

Reviewer Expertise:

Drug delivery, polymer

I confirm that I have read this submission and believe that I have an appropriate level of expertise to confirm that it is of an acceptable scientific standard.

## References

[1] : Recent trends on wound management: New therapeutic choices based on polymeric carriers. *Asian J Pharm Sci* .2020;15(6) : 10.1016/j.ajps.2019.11.008 661-684 10.1016/j.ajps.2019.11.008 33363624PMC7750807
